# Association between Physical Frailty Subdomains and Oral Frailty in Community-Dwelling Older Adults

**DOI:** 10.3390/ijerph18062931

**Published:** 2021-03-12

**Authors:** Ryo Komatsu, Koutatsu Nagai, Yoko Hasegawa, Kazuki Okuda, Yuto Okinaka, Yosuke Wada, Shotaro Tsuji, Kayoko Tamaki, Hiroshi Kusunoki, Hiromitsu Kishimoto, Ken Shinmura

**Affiliations:** 1 Department of Physical Therapy, School of Rehabilitation, Hyogo University of Health Sciences, 1-3-6 Minatojima, Chuo-ku, Kobe, Hyogo 650-8530, Japan; pr17024@std.huhs.ac.jp (R.K.); pr17012@std.huhs.ac.jp (K.O.); pr17011@std.huhs.ac.jp (Y.O.); 2 Department of Dentistry and Oral Surgery, Hyogo College of Medicine, 1-1 Mukogawa-cho, Nishinomiya, Hyogo 663-8501, Japan; cem17150@dent.niigata-u.ac.jp (Y.H.); kisihiro@hyo-med.ac.jp (H.K.); 3 Division of Comprehensive Prosthodontics, Faculty of Dentistry, Graduate School of Medical and Dental Sciences, Niigata University, Niigata 951-8514, Japan; 4 Department of Rehabilitation, Hyogo College of Medicine Sasayama Medical Center, Sasayama, Hyogo 669-2321, Japan; yu-wada@hyo-med.ac.jp; 5 Department of Orthopaedic Surgery, Hyogo College of Medicine, Nishinomiya, Hyogo 663-8501, Japan; tj13041305sho@gmail.com; 6 Department of General Medicine, Hyogo College of Medicine, 1-1 Mukogawa, Nishinomiya, Hyogo 663-8501, Japan; ka-tamaki@hyo-med.ac.jp (K.T.); kusunoki1019@yahoo.co.jp (H.K.); ke-shimmura@hyo-med.ac.jp (K.S.)

**Keywords:** physical frailty, oral frailty, physical performance, older adults

## Abstract

This cross-sectional study aimed to demonstrate the association between physical frailty subdomains and oral frailty. This study involved community-dwelling older adults (aged ≥65 years). Physical frailty was assessed with the Japanese version of the Cardiovascular Health Study criteria. Oral frailty was defined as limitations in at least three of six domains. Logistic regression analysis was used to analyze the association between physical frailty risk and oral frailty. In addition, we examined the association between physical frailty subdomains (gait speed, grip strength, exhaustion, low physical activity, and weight loss) and oral frailty. A total of 380 participants were recruited for this study. Overall, 18% and 14% of the participants were at risk of physical frailty and had oral frailty, respectively. Physical frailty risk (odds ratio (OR) = 2.40, 95% confidence interval (CI): 1.22–4.75, *p* = 0.012) was associated with oral frailty in multivariate analysis. In secondary analysis, among physical frailty subdomains, gait speed (OR = 0.85, 95% CI: 0.73–0.97, *p* = 0.019) was associated with oral frailty. The present findings suggest that physical frailty is closely related to oral frailty. Among physical frailty subdomains, decreased gait speed in particular is an important indicator related to the development of oral frailty.

## 1. Introduction

According to the Fried phenotype, physical frailty is defined as physical and functional vulnerability [1]. Physical frailty includes low gait speed, weakness, exhaustion, low physical activity, and weight loss; as these domains are interrelated, a decline in one tends to drive a decline in another [2]. Physical frailty increases the risk of long-term care needs, falls, premature mortality, hospitalization, and disability [1,3,4]. Consequently, maintaining and improving overall health is important to preventing frailty and the associated consequences.

In addition, oral frailty is an important indicator of overall health. Oral frailty is defined as poor oral status that increases the risk of physical frailty, sarcopenia, long-term care needs, and premature mortality [5]. Moreover, oral frailty has been associated with social frailty and malnutrition, both of which increase the risk of physical frailty [6,7,8,9,10,11]. This evidence suggests that oral frailty is a significant contributor to health and thus a target for prevention.

A previous study has demonstrated that oral frailty may affect physical frailty [5]. Meanwhile, a previous study has shown that skeletal muscle mass index and grip strength among women might predict dysphagia [12]. In this way, physical and oral frailty may be associated with each other. This finding suggests that an intervention to improve physical function may ameliorate oral function.

In the present study, we aimed to examine the relationship between physical frailty and oral frailty. Although previous studies have shown a relationship between several domains of physical frailty (grip strength, gait speed, and physical activity) and oral health status (tongue pressure and chewing ability) [13,14], the impact of each physical frailty subdomain on overall oral health remains unclear. The physical frailty subdomains are independent concepts; therefore, clarifying the association between physical frailty subdomains and oral frailty will facilitate the development of interventions corresponding to each domain. Herein, we aimed to clarify the relationship between physical frailty subdomains and oral frailty to provide evidence that may be used to interject the interrelationship between oral and physical health decline.

## 2. Materials and Methods

This cross-sectional study involved community-dwelling older adults living in the Sasayama-Tamba Area, which is a rural area in the Hyogo prefecture, Japan. The population of Tamba-Sasayama city was 41,490 as of 2015; the average age tends to be higher in this prefecture than in Japan overall (31.4% of the population were aged ≤65 years as of September 2015). Tamba-Sasayama City is located in the mountainous region of the Hyogo prefecture, where the leading sectors remain farming and agriculture. Data used in the present study were collected between April 2016 and December 2019. To recruit participants, we used newspaper inserts and poster advertisements at the Hyogo College of Medicine Sasayama Medical Center. We included individuals aged ≥65 years. Exclusion criteria were individuals who (1) had missing data, and (2) had decreased cognitive function (Mini-Mental State Examination (MMSE) score <20) [15]. The data used in this study were anonymized. This study was approved by the institutional review board of the Hyogo College of Medicine (No. Rinhi 0342). All participants took part in this study voluntarily and provided written informed consent to be included. This study was conducted in compliance with the Helsinki Declaration.

### 2.1. Participant Characteristics

We assessed the following characteristics: age, height, body mass index (BMI), number of prescribed medications, comorbidities, cognitive function, education, and social activity. BMI was calculated by dividing body weight (kg) by the height (m) squared. Cognitive function was assessed using the MMSE. Social activity was evaluated by a response of “yes” or “no” using the following question from the Japan Science and Technology Agency Index of Competence [16]: “Do you participate in any community activities or volunteer activities?”

### 2.2. Physical Frailty

We considered physical frailty risk by limitations in at least two of the following conditions, the reference to the Japanese version of the Cardiovascular Health Study (J-CHS): [17] low gait speed, weakness, exhaustion, low physical activity, and weight loss. To assess gait speed, we asked participants to walk a 12 m walkway at their normal speed and measured the time for a 10 m walk. We established slow gait speed according to a cutoff point of <1.0 m/s [18]. Weakness was assessed using maximum grip strength, which was measured in kilograms using a Smedley-type handheld dynamometer (GRIP-A; Takei Ltd., Niigata, Japan). Weakness was established according to a sex-specific cutoff (<26 kg for men and <18 kg for women) [19]. We considered exhaustion if participants responded “yes” to the following question from the Kihon Checklist (KCL) developed by the Japanese Ministry of Health, Labor, and Welfare (Supplementary Table S1) [20]: “In the last 2 weeks, have you felt tired without a reason?” Low physical activity was considered if participants responded “no” for the given question: [18] “Do you engage in low levels of physical exercise aimed at health?” Weight loss was assessed by a response of “yes” to the question, “Have you lost 2 kg or more in the past 6 months?” [20].

### 2.3. Oral Frailty and Related Assessments

We defined oral frailty as functional limitations in at least three of the following six domains [5]: few remaining teeth, reduced masticatory performance, reduced articulatory oral motor skills (examined using the ability to pronounce the “ta” sound), lower maximum tongue pressure, subjective difficulty eating hard foods, and subjective difficulty swallowing. The number of corresponding items was used as the oral frailty score. Participants whose oral frailty score was two or fewer were considered to be without oral frailty.

The number of remaining teeth was assessed by dentists. The cutoff value for few remaining teeth was of <20 [5]. We used a previously described scoring method to assess low masticatory performance [21]. Participants were instructed to chew a piece of test gummy jelly (UHA Mikakuto Co., Ltd., Osaka, Japan) freely 30 times; subsequently, the pieces of gummy jelly were objectively evaluated by a visual scoring method (on a scale from 0–9 points). The cutoff value for low masticatory performance was a score of ≤2 points [22]. Articulatory oral motor skills (pronunciation of the “ta” sound) were assessed using oral diadochokinesis (“ta”). Participants were asked to pronounce each syllable consecutively as fast as possible for 5 s. We measured articulation counts per second using a digital counter (KENKOU-KUN^®^; Takei Scientific Instruments Co., Ltd., Niigata, Japan). We established sex-specific cutoff values for oral diadochokinesis (“ta”), which were equivalent to <5.2 times/s and <5.4 times/s for men and women, respectively [5].

Lower maximum tongue pressure was measured at the midline of the dorsal surface of the tongue using a tongue pressure measurement device (JMS tongue pressure meter, JMS, Hiroshima, Japan). We established a sex-specific cutoff value for the lower maximum tongue pressure (<27.4 kPa and 26.5 kPa for men and women, respectively) [5]. We considered subjective difficulty in eating hard foods to be present in participants that responded “yes” to the following question from KCL (Supplementary Table S1) [20]: “Compared to 6 months ago, do you find it more difficult to eat hard foods?” Subjective difficulty in swallowing was considered present in participants that responded “yes” to the following question: “Do you choke on tea or soup?” [20].

In addition to the oral frailty criteria, occlusal support was assessed using the Eichner classification, which was divided into 10 levels (A1–C3) according to the number of occlusal teeth pairs (the A1 level contained most pairs) [23].

### 2.4. Statistics

Continuous variables were assessed for the normality of distribution using the Shapiro–Wilk test. We used the Mann–Whitney U test to compare age, height, BMI, number of medications, the MMSE scores, education level, gait speed, and grip strength between oral robust and frailty. Differences in sex, comorbidities, history of falls, social activity levels, physical frailty risk, and physical frailty subdomains between the groups were assessed using the χ^2^ test.

We conducted two types of analysis using oral frailty as the dependent variable. Logistic regression analysis was used to analyze the association between physical frailty risk and oral frailty. Physical frailty risk as the independent variable was included in the crude model. Further, we included age, gender, the MMSE score, BMI, number of medications, and number of comorbidities as covariates, provided they were significantly associated with oral function in univariate models; we used the forced entry method in the adjusted model. In addition, to examine the relationship between components of physical frailty and oral frailty, we conducted an analysis including five physical frailty subdomains as independent variables, following J-CHS: gait speed, grip strength, exhaustion, low physical activity, and weight loss. We aimed to explore the factors of the physical frailty subdomain associated with oral frailty; hence, we included these as independent variables using the stepwise method in the crude model. Gait speed and grip strength were used as continuous values. The covariates (those defined in the first analysis) were included using the forced entry method in the adjusted model.

All analyses were performed with IBM SPSS version 24 (IBM Japan Ltd., Tokyo, Japan). *p*-Values of < 0.05 were considered indicative of statistically significant findings.

## 3. Results

A total of 382 community-dwelling older adults who met the eligibility criteria were included in this study. Two participants lacked data required for multivariate analysis and were thus excluded. The final sample included 380 participants (116 men; mean age 72.8 ± 5.6 years). Sixty-nine (18%) and 54 (14%) participants had physical frailty risk and oral frailty, respectively (Table 1). The prevalence of low gait speed, weakness, exhaustion, low physical activity, and weight loss was 18 (4.7%), 24 (6.3%), 76 (20%), 134 (35%), and 54 (14%), respectively. Nineteen (35%) older adults with oral frailty showed physical frailty risk. The prevalence of oral frailty in each physical frailty subdomain was 6 (11%) (*p*-value for the difference between the oral robust and frailty group = 0.017), 5 (9.3%) (*p* = 0.337), 18 (33%) (*p* = 0.008), 26 (48%) (*p* = 0.032), and 12 (22%) (*p* = 0.069) for low gait speed, weakness, exhaustion, low physical activity, and weight loss, respectively. There were significant between-group differences in age (*p* < 0.001), cardiovascular disease prevalence (*p* = 0.002), education level (*p* = 0.038), social activity level (*p* = 0.002), and gait speed (*p* = 0.001) between participants with oral robust and with oral frailty.

The prevalence of each oral frailty subdomain was as follows: 100 (26%), 73 (19%), 63 (17%), 77 (20%), 63 (17%), and 84 (22%) for few remaining teeth, low masticatory performance, low articulatory oral motor skill (pronunciation of the “ta” sound), lower maximum tongue pressure, subjective difficulty in eating hard foods, and subjective difficulty swallowing, respectively (Table 2).

The prevalence of each oral frailty subdomain among older adults with oral frailty was as follows: 43 (80%), 37 (69%), 24 (44%), 24 (44%), 33 (61%), and 29 (54%) for few remaining teeth, low masticatory performance, low articulatory oral motor skill (pronunciation of the “ta” sound), lower maximum tongue pressure, subjective difficulty in eating hard foods, and subjective difficulty swallowing, respectively. Eichner classifications were significantly different between the oral robust and frailty groups (*p* < 0.001).

Logistic regression analysis revealed that physical frailty risk (odds ratio (OR) = 3.00, 95% confidence interval (CI): 1.59–5.65, *p* = 0.001) was associated with oral frailty in model 1 (Table 3). In model 2 (adjusted model), physical frailty risk (OR: 2.40, 95% CI: 1.22–4.75, *p* = 0.012) remained significantly associated with oral frailty.

In the analysis of oral and physical frailty subdomains, gait speed (OR = 0.80, 95% CI: 0.70–0.92, *p* = 0.001) and exhaustion (OR = 2.19, 95% CI: 1.15–4.17, *p* = 0.017) were significantly associated with oral frailty in model 1 (Table 3). Significant relationships in model 1 were not detected in weakness, low physical activity, and weight loss with oral frailty. In model 2 (adjusted model), gait speed (OR = 0.85, 95% CI: 0.73–0.97, *p* = 0.019) remained significantly associated with oral frailty. Exhaustion was not significantly associated with oral frailty in model 2.

## 4. Discussion

In this study, we investigated the association between physical and oral frailty. In addition, we attempted to identify physical frailty subdomains that were significantly associated with oral frailty. In the present study, physical frailty risk was associated with oral frailty. Moreover, gait speed, a physical frailty subdomain, was associated with oral frailty.

The prevalence of oral frailty was 14% in the present study, which was similar to that previously reported [5,6,7]. Among the subdomains of oral frailty, the item representing few remaining teeth was the most prevalent. Factors associated with tooth loss include periodontal disease, dental caries, prosthodontic treatment, age, low socioeconomic status, diabetes mellitus, mean initial bone loss, and smoking [24,25,26]. Among these factors, periodontal disease and dental caries are considered major risk factors for tooth loss [27]. Compared to other subdomains in oral frailty, having few remaining teeth was the most prevalent (80%), which suggests that prioritizing tooth loss prevention may be a key to avert the development of oral frailty.

The present study has shown an association between physical frailty risk and oral frailty. A previous study reported higher prevalence of oral frailty among older adults with physical pre-frailty signs than among those without such signs [6]. A longitudinal study by Tanaka et al. has shown that oral frailty is a risk factor for the future development of physical frailty [5]. In addition to studies that investigated the overall relationship between oral and physical function using the frailty concept, several studies have demonstrated relationships between physical (grip strength, gait speed, and physical activity) and oral (tongue pressure and chewing ability) frailty components [13,14]. The present study findings are consistent with those of these previous studies, providing additional evidence for the relationship between oral and physical frailty.

Gait speed, a subdomain of physical frailty, was significantly associated with oral frailty. Oral frailty may cause physical frailty, as the former may affect nutritional status among older adults and lead to weight loss [8]. Xue et al. have suggested a frailty cycle model, whereby weight loss leads to weakness, exhaustion, and low gait speed [2]. In the present study, weight loss and grip strength between the oral robust and frailty groups were not significantly different even in the univariate analysis (weight loss: *p* = 0.069, grip strength: *p* = 0.055). Since lower gait speed was the only significant factor, it is difficult to determine the relationship between other subdomains (weight loss, weakness, fatigue, and muscle weakness) and oral frailty in this study. Further research is needed on the relationship between the frailty cycle and oral frailty. Additionally, the fact that only lower gait speed was statistically significant suggests that oral frailty may be a sensitive indicator of declined gait speed. Meanwhile, tooth loss may accelerate the decline of gait speed [28]. However, this pathway is likely complex and hierarchical and thus difficult to assess in a cross-sectional design. Nevertheless, the present result suggests the necessity to assess both oral function and gait performance in clinical practice.

These findings notwithstanding, the association between physical and oral frailty may have the reverse direction. Lower gait speed is a risk factor for social frailty, whereby limited mobility may reduce the likelihood of social interactions [29]. Social frailty, which may include reduced social activity or contact with other people, may reduce opportunities for verbal communications among older adults, which in turn may affect oral function as a result of the reduced use of the muscles surrounding the pharynx and mouth [30]. In the present study, a significant difference in social activity was observed between the oral robust and frailty groups in the univariate analysis, indicating a possibility that low gait speed may increase the risk of oral frailty. Longitudinal studies are required to elucidate the causal relationship between physical and oral frailty.

This study has three limitations. First, it was a cross-sectional study, which precluded any meaningful discussions of causality. Second, the present participants volunteered to be included in this study, which may have resulted in selection bias. Third, in this study the criterion for physical frailty was operationally defined as two or more items being applicable, referring to the criteria of previous studies. Therefore, it is different from the more commonly used definition of three or more items, which may limit the generalizability of results. Incidentally, in the present study, masticatory performance assessment was conducted using a different material from that of the previous study. Gummy jelly assesses chewing ability, as well as chewing gum. Meanwhile, an earlier study reported that chewing gum and gummy jelly could assess different types of ability, with bias toward mixing and shearing ability, respectively [31]. Although the properties of the two materials are strictly different, we assumed that the differences did not significantly influence the results from the perspective of assessing the same ability in a broad sense. These limitations notwithstanding, this is the first study to examine the relationship between oral frailty and physical frailty subdomains. Knowing these relationships is important to prevent and treat frailty in clinical settings.

## 5. Conclusions

In this study, physical frailty risk was significantly associated with oral frailty. In addition, lower gait speed (a physical frailty subdomain) was associated with increased prevalence of oral frailty. Further studies are required to clarify the causal relationship between physical and oral frailty to develop suitable prevention and treatment interventions.

## Figures and Tables

**Table 1:** Characteristics in participants with oral frailty.

Variables	Overall (*n* = 380)	Oral Robust (*n* = 326)	Oral Frailty (*n* = 54)	*p*-Value
Age, years, mean (SD)	72.8 (5.5)	72.2 (5.3)	76.4 (6.0)	<0.001
Female, *n* (%)	264 (69)	224 (69)	40 (74)	0.428
Height, cm, mean (SD)	155.5 (8.3)	155.8 (8.2)	153.6 (8.5)	0.069
Body mass index, kg/m^2^, mean (SD)	22.9 (2.9)	22.9 (2.9)	23.0 (3.0)	0.854
Medication, *n*, median (IQR)	1.5 (0–3)	1.0 (0–3)	2.0 (0–4)	0.068
Comorbidities, *n* (%)				
Hypertension	170 (45)	142 (44)	28 (52)	0.256
Diabetes	44 (12)	38 (11)	6 (11)	0.919
Kidney disease	18 (4.7)	16 (4.9)	2 (3.7)	0.700
Cardiovascular disease	26 (6.8)	17 (5.2)	9 (17)	0.002
Osteoporosis	50 (13)	42 (13)	8 (15)	0.697
MMSE, median (IQR)	29 (27–30)	29 (27–30)	28 (27–30)	0.436
Education, years, median (IQR)	12 (12–14)	12 (12–14)	12 (9–14)	0.038
Social activity, *n* (%)	102 (27)	78 (24)	24 (44)	0.002
Physical frailty, *n* (%)				<0.001
Robust	311 (82)	278 (85)	33 (65)	
Frailty risk (>2 points)	69 (18)	50 (15)	19 (35)	
Physical frailty subdomains, *n* (%)				
Low gait speed	18 (4.7)	12 (3.7)	6 (11)	0.017
Weakness	24 (6.3)	19 (5.8)	5 (9.3)	0.337
Exhaustion	76 (20)	58 (18)	18 (33)	0.008
Low physical activity	134 (35)	108 (33)	26 (48)	0.032
Weight loss	54 (14)	42 (13)	12 (22)	0.069
Gait speed, m/s, mean (SD)	1.4 (0.2)	1.5 (0.2)	1.3 (0.2)	0.001
Grip strength, kg, mean (SD)	27.3 (8.0)	27.7 (7.7)	25.4 (9.0)	0.055

SD, standard deviation; IQR, interquartile range; MMSE, Mini-Mental State Examination. χ^2^ test for proportions and nominal variables, Mann–Whitney U test for nonparametric variables.

**Table 2:** Distribution of participants per oral functions and statuses.

Variables	Overall (*n* = 380)	Oral Robust (*n* = 326)	Oral Frailty (*n* = 54)	*p*-Value
Oral frailty subdomains, *n* (%)				
Few remaining teeth	100 (26)	57 (18)	43 (80)	<0.001
Low masticatory performance	73 (19)	36 (11)	37 (69)	<0.001
Low articulatory oral motor skill (pronunciation of “ta” sound)	63 (17)	39 (12)	24 (44)	<0.001
Lower maximum tongue pressure	77 (20)	53 (16)	24 (44)	<0.001
Subjective difficulty in eating hard foods	63 (17)	30 (9.2)	33 (61)	<0.001
Subjective difficulty in swallowing	84 (22)	55 (17)	29 (54)	<0.001
Eichner classification, *n* (%)				<0.001
A1	58 (15)	58 (18)	0 (0)	
A2	81 (21)	77 (24)	4 (7)	
A3	46 (12)	45 (14)	1 (2)	
B1	62 (16)	58 (18)	4 (7)	
B2	43 (11)	37 (11)	6 (11)	
B3	32 (8)	20 (6)	12 (22)	
B4	15 (4)	10 (3)	5 (9)	
C1	7 (2)	5 (2)	2 (4)	
C2	22 (6)	10 (3)	12 (22)	
C3	14 (4)	6 (2)	8 (15)	

χ^2^ test for proportions and nominal variables, Mann–Whitney U test for ordinal variables.

**Table 3:** Relationship between physical and oral frailty.

	Model 1			Model 2		
	OR	95% CI	*p*-Value	OR	95% CI	*p*-Value
Overall (*n* = 380)						
Physical frailty risk	3.00	1.59–5.65	0.001	2.40	1.22–4.75	0.012
Physical frailty subdomains						
Exhaustion	2.19	1.15–4.17	0.017	1.93	0.96–3.87	0.062
Gait speed	0.80	0.70–0.92	0.001	0.85	0.73–0.97	0.019

Model 1 was a crude model without any adjustments. Physical frailty subdomains were added as independent variables in stepwise analysis. Model 2 was adjusted for age, gender, cardiovascular disease, MMSE, body mass index (BMI), and number of medications prescribed, using the forced entry method. OR, odds ratio.

## Data Availability

The data that support the findings of this study are available from the corresponding author, upon reasonable request.

## Supplementary Materials

The following are available online at https://www.mdpi.com/1660-4601/18/6/2931/s1, Table S1: Kihon Checklist English version created by Japan Geriatrics Society Working Group on Frailty.
